# New arylsparteine derivatives as positive inotropic drugs

**DOI:** 10.1080/14756366.2017.1279156

**Published:** 2017-01-29

**Authors:** Vito Boido, Marcella Ercoli, Michele Tonelli, Federica Novelli, Bruno Tasso, Fabio Sparatore, Elena Cichero, Paola Fossa, Paola Dorigo, Guglielmina Froldi

**Affiliations:** a Department of Pharmacy, University of Genoa, Genoa, Italy;; b Department of Pharmaceutical and Pharmacological Sciences, University of Padova, Padova, Italy

**Keywords:** Lupin alkaloids, 2-aryl-2-dehydrosparteines, 2-arylsparteines, positive inotropic agents

## Abstract

Positive inotropic agents are fundamental in the treatment of heart failure; however, their arrhythmogenic liability and the increased myocardial oxygen demand strongly limit their therapeutic utility. Pursuing our study on cardiovascular activities of lupin alkaloid derivatives, several 2-(4-substituted-phenyl)-2-dehydrosparteines and 2-(4-substituted-phenyl)sparteines were prepared and tested for inotropic and chronotropic activities on isolated guinea pig atria. Four compounds (**6b**, **6e**, **7b**, and **7f**) exhibited significant inotropism that, at the higher concentrations, was followed by negative inotropism or toxicity. Compound **7e** (2-(4-tolyl)sparteine) exhibited a steep dose-depending inotropic activity up to the highest concentration tested (300 µM) with an *E*_max_ of 116.5 ± 3.4% of basal force, proving less potent but much more active in comparison to the highest concentrations tested of digoxin and milrinone having *E*_max_ of 87.5 ± 3.1% and 52.2 ± 1.1%, respectively. Finally, docking studies suggested that the relevant sparteine derivatives could target the sigma-1 receptor, whose involvement in cardiac activity is well documented.

## Introduction

Congestive heart failure (CHF) is a chronic and progressive disorder of left ventricular myocardial remodeling, which may lead to acute decompensation, resulting in a major cause of death in patients with heart disease^1^. To relieve symptoms and to improve cardiac function, several classes of drugs are available, such as diuretics, ACE inhibitors, angiotensin receptor blockers, β-adrenergic receptor antagonists, and others. To increase the impaired contractile ability of heart, digitalis glycosides (digoxin and others) are used since long time, while β-adrenergic agonists (ibopamine and dobutamine) and phosphodiesterase III inhibitors (inamrinone, milrinone, enoximone, and others) have been found to be more profitable than digoxin in some clinical circumstances^2–4^. Unfortunately, these drugs are not tolerated for long-term therapy and may even exacerbate the myocardial abnormality with disappointing effect on mortality rate.

Thus, despite the narrow therapeutic index, digoxin has been re-appraised, but better tolerated inotropics, possibly acting through novel mechanisms, with reduced myocardial oxygen demand and lower arrhythmogenic liability are actively pursued. Indeed, in the last years, novel chemotypes and biological targets have been investigated producing a number of promising compounds, such as istaroxime, ivabradine, levosimendan, and omecamtiv mecarbil, which are described in several reviews^5–10^.

Some other compounds, among those displaying positive inotropic activity, appear to have been somewhat underestimated. In fact, even possessing only moderate inotropic activity, they target different heart structures with antiarrhythmic and/or coronaro-dilator effects. Particularly, we deemed of interest the family of lupin (quinolizidine) alkaloids, whose pharmacophoric template has been only limitedly explored for drug development.

**Figure 1.**
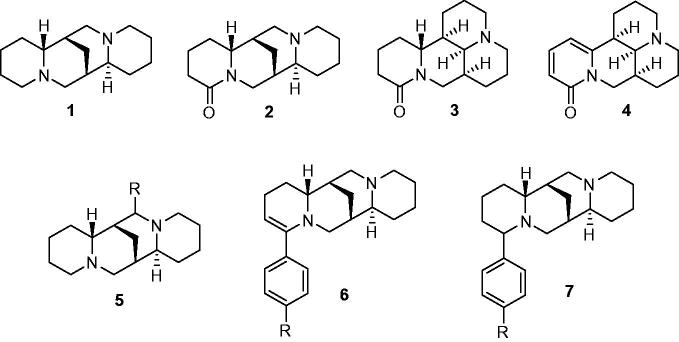
Lupin alkaloids and derivatives investigated as inotropic agents.

Positive inotropic activity, besides antiarrhythmic action, was observed for (−)-sparteine (**1**), (±)-lupanine (**2**) (Figure 1), and other structurally related alkaloids11a–11d in isolated atria of rat or guinea pig; however, the positive inotropic action was not confirmed in the intact heart of anesthetized dog11c^,^^12^. Particularly, Philipsborn et al.11b reported for sparteine sulfate, in the range from 1.6 µM to 1.6 mM concentration, a gradual increase of the inotropic activity in electrically driven guinea pig left atria, with a maximum increase of 22% of contractile force at 100 µM concentration; no variation was observed up to 100 µM concentration, in the case of spontaneously beating right atria. Beyond 100 µM concentration, a steep decrease of activity was observed in both kinds of atria preparations, reaching −70% at the highest tested concentration.

Direct positive inotropic action on isolated cardiac muscle of guinea pig and bullfrog was described for another kind of tetracyclic lupin alkaloids, as (+)-matrine (**3**) and (−)-sophoramine (**4**)^13^ (Figure 1).

In order to improve the cardiovascular activity of sparteine, some modifications of its structure have been also explored. A set of 17-alkylsparteines (from 17-methyl to 17-hexylsparteine) (**5**, R = alkyl) showed improved inotropic effects in electrically stimulated guinea pig atria11d^,^^14^. According to Engelmann et al.11d, in electrically driven guinea pig atria, sparteine produced a maximal increase (+42%) of contractile force at 200 µM concentration, while the 17-ethylsparteine, at the same concentration, displayed a +60% increase that reached +81% at the highest tested concentration (800 µM), resulting the most active 17-alkylsparteine derivative as inotropic. On the contrary, 17-butyl and 17-pentylsparteines were the best as antifibrillatory agents^14^. The replacement of the 17-alkyl substituents with heteroaryl-, heteroarylalkyl- and benzyl-moieties led to compounds with antiarrhythmic activity and reduced oxygen consumption by myocardium, so that 17-(3-methoxybenzyl)sparteine (**5**, R= 3-methoxybenzyl) was suggested for the therapy of coronary insufficiency15a^,^15b (Figure 1).

Some 2-alkyl- and 2-arylderivatives of 2-dehydrosparteine (**6**, R = H, OCH_3_) and sparteine (**7**, R = H, OCH_3_) were recognized to display 10- to 30-fold higher antifibrillatory activity on isolated frog heart in comparison to sparteine^16^^,^^17^, but the eventual inotropic activity was not investigated (Figure 1).

**Figure 2.**
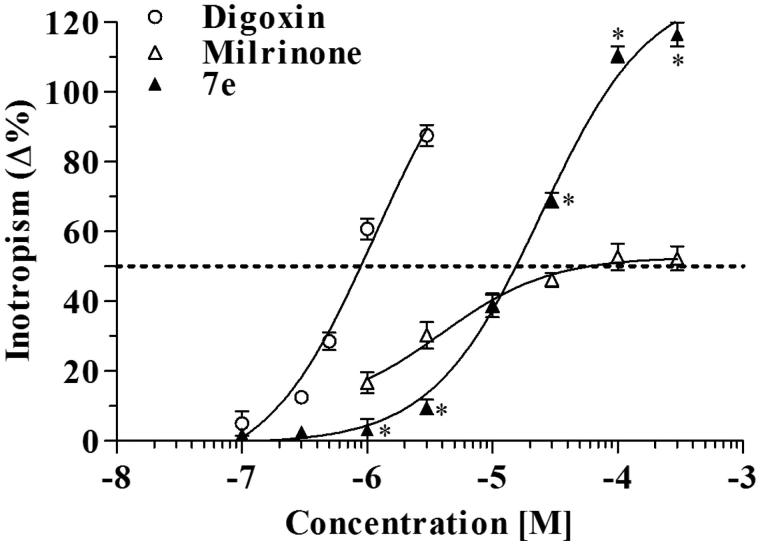
Inotropic effects of digoxin, milrinone and compound **7e**. *p < 0.05 compound **7e** versus milrinone.

Finally, through a pharmacological screening of assorted quinolizidine derivatives performed, some years ago, by “Panlabs Inc.” (Bothell, WA), we observed that the 2-(4-fuorophenyl)sparteine displayed a significant positive inotropic activity on electrically driven guinea pig atria. Therefore, pursuing our long standing study about cardiovascular activity of derivatives of lupin alkaloids^18–22^, we deemed interesting to extend the study of inotropic activity to twelve 2-aryl-2-dehydrosparteine and 2-arylsparteine of structures **6** and **7**, respectively, with R = H, F, Cl, OCH_3_, CH_3_, and CF_3_ (Figure 2), thus including the four compounds previously investigated by Winterfeld et al.^16^^,^^17^ for the antifibrillatory activity.

## Materials and methods

### General

Chemicals, solvents, and reagents used for the syntheses were purchased from Sigma-Aldrich (St. Louis, MO), Fluka (Newport News, VA), or Alfa Aesar (Ward Hill, MA), and were used without any further purification. Column chromatography (CC): silica gel (Merck, Darmstadt, Germany). Mps: Büchi apparatus (Büchi, Flawil, Switzerland), uncorrected. ^1^H NMR and ^13^C NMR spectra: Varian Gemini-200 spectrometer (Varian Medical Systems (VAR), Palo Alto, CA); CDCl_3_; *δ* in ppm rel. to Me_4_Si as an internal standard; *J* in Hz. Elemental analyses were performed on a Carlo Erba EA-1110 CHNS-O instrument in the Microanalysis Laboratory of the Department of Pharmacy of Genoa University.

#### Extraction of (±)-lupanine

The ground seeds of *Lupinus albus L.* (1 kg) were extracted with light petroleum ether in a Soxhlet apparatus for 24 h to eliminate the lipidic materials. Successively the air dried material was further extracted with methanol for about 36 h. The methanol solution was filtered and concentrated to small volume in a Büchi Rotavapor (Büchi, Flawil, Switzerland). To the concentrated extract, 300 mL of water followed by 150 mL of 2 N hydrochloric acid were added and the acidic solution was filtered and extracted three times with ether to eliminate all non-basic compounds. The acidic solution was basified with 6 N sodium hydroxide solution and extracted with chloroform (5 × 100 mL). After drying (Na_2_SO_4_), the chloroform was evaporated in Rotavapor obtaining 22.7 g of an oil that partially crystallized standing in cold. The addition of a little of acetone allowed the filtration of the crystals that were recrystallized from acetone yielding 8.3 g of pure (±)-lupanine melting at 95–97 °C. The joined acetone solution was evaporated to dryness and the residue was chromatographed on basic alumina (220 g) eluting with chloroform (15 × 40 mL). The elimination of the solvent left 6.65 g of crystals that were rinsed with a little of acetone yielding 5.5 g of (±)-lupanine with m.p. 93–94 °C. Therefore, the total yield of (±)-lupanine was 1.38% in respect to the seeds used.

#### 2-(4-Substituted-phenyl)-2-dehydrosparteines (6). (General method)

A solution of aryl magnesium bromide (20.1 mmol) was prepared by reacting at r.t. Mg turnings (0.51 g, 21.0 mmol) in dry Et_2_O (10 mL), activated by methyl iodide and iodine, with a solution of the proper aryl bromide (20.1 mmol) in dry Et_2_O (10 mL). Then a solution of lupanine (2 g, 8.1 mmol) in dry Et_2_O (50 mL) was added. After being refluxed for 2 h, to the cooled (0–5 °C) reaction mixture, 50 mL of a solution of 2N HCl were added, then the resulting mixture was washed with Et_2_O in order to remove the aromatic compounds derived from the exceeding arylmagnesium bromides. The acidic solution was basified with a solution of 6N NaOH and extracted with Et_2_O. The dried organic layer (Na_2_SO_4_) was evaporated, leaving an oily residue that was purified by CC(SiO_2_/Et_2_O + 2%DEA) and, when necessary, crystallized from the proper solvent.

##### 2-Phenyl-2-dehydrosparteine (6a)

Yield: 35%. Mp 99–100 °C (acetone) [lit. (16a): 103–105 °C]. ^1^H NMR (200 MHz, CDCl_3_): 1.10–2.48 (m, 18 H), 2.70–3.04 (m, 4 H), 4.41–4.53 (m, 1 H, C(3)), 7.18–7.43 (m, 5 H, ArH). Anal. Calcd for C_21_H_28_N_2 _+_ _0.25H_2_O: C, 80.59; H, 9.18; N, 8.95. Found: C, 80.71; H, 9.14; N, 8.77.

##### 2-(4’-Fluorophenyl)-2-dehydrosparteine (6b)

Yield: 49%. Mp 124–125 °C (Et_2_O). ^1^H NMR (200 MHz, CDCl_3_): 1.10–2.47 (m, 18 H), 2.70–2.95 (m, 4 H), 4.44–4.56 (m, 1 H, C(3)), 6.92–7.07 (m, 2 H, ArH), 7.21–7.37 (m, 2 H, ArH). ^13^C NMR (50 MHz, CDCl_3_): 163.18, 158.31, 146.90, 135.27, 128.38, 101.33, 63.39, 61.27, 54.68, 53.66, 51.81, 35.06, 33.34, 31.89, 26.67, 25.81, 24.76, 23.96, 21.70. Anal. Calcd for C_21_H_27_FN_2_: C, 77.26; H, 8.34; N, 8.58. Found: C, 77.21; H, 8.35; N, 8.49.

##### 2-(4’-Chlorophenyl)-2-dehydrosparteine (6c*)*

Yield: 37%. Mp 130–133 °C (Et_2_O). ^1^H NMR (200 MHz, CDCl_3_): 1.09–2.40 (m, 18 H), 2.68–2.94 (m, 4 H), 4.46–4.57 (m, 1 H, C(3)), 7.27 (pseudo s, 4 H, ArH). ^13^C NMR (50 MHz, CDCl_3_): 146.84, 137.75, 131.41, 128.10, 127.06, 101.77, 63.38, 61.27, 54.67, 53.71, 51.77, 35.03, 33.34, 31.87, 26.66, 25.74, 24.75, 23.95, 21.72. Anal. Calcd for C_21_H_27_ClN_2_: C, 73.56; H, 7.92; N, 8.17. Found: C, 73.22; H, 8.01; N, 8.11.

##### 2-(4’-Methoxyphenyl)-2-dehydrosparteine (6d*)*

Yield: 38%. Oil (lit. (16b): oil, b.p. 194–202 °C, high *vacuum*). ^1^H NMR (200 MHz, CDCl_3_): 1.00–3.35 (m, 22 H), 3.88 (s, OCH_3_), 4.45–4.67 (m, 1 H, C(3)), 6.92 (d, *J* = 10.0 Hz, 2 H, ArH), 7.36 (d, *J* = 10.0 Hz, 2 H, ArH). Elemental analysis produced results not well fitting for the formula C_22_H_30_N_2_O, due to the instability of the compound.

##### 2-(4’-Tolyl)-2-dehydrosparteine (6e)

Yield: 39%. Mp 102–103 °C (acetone) [(lit.^23^: 113–115 °C]. ^1^H NMR (200 MHz, CDCl_3_): 1.09–2.44 (m, 18 H and 2.34, s, CH_3_, superimposed), 2.68–3.03 (m, 4 H), 4.46–4.56 (m, 1 H, C(3)), 7.12 (d, *J* = 9.8 Hz, 2 H, ArH), 7.23 (d, *J* = 9.8 Hz, 2 H, ArH). ^13^C NMR (50 MHz, CDCl_3_): 147.85, 136.40, 135.36, 127.56, 126.73, 100.68, 63.28, 61.27, 54.68, 51.85, 35.09, 33.40, 31.98, 26.73, 25.88, 24.84, 23.97, 21.76, 20.10. Anal. Calcd for C_22_H_30_N_2_: C, 81.94; H, 9.38; N, 8.69. Found: C, 82.15; H, 9.45; N, 8.70.

##### 2-(4’-Trifluoromethyl)-2-dehydrosparteine (6f)

Yield: 28%. Mp 98–99 °C (acetone). ^1^H NMR (200 MHz, CDCl_3_): 1.05–2.47 (m, 18 H), 2.69–3.03 (m, 4 H), 4.46–4.54 (m, 1 H, C(3)), 7.44 (d, *J* = 9.2 Hz, 2 H, ArH), 7.58 (d, *J* = 9.0 Hz, 2 H, ArH). ^13^C NMR (50 MHz, CDCl_3_): 146.87, 142.96, 127.56, 126.98, 123.87, 102.98, 63.46, 61.27, 54.70, 53.78, 51.73, 34.99, 33.23, 31.83, 26.61, 25.67, 24.67, 23.92, 21.77. Anal. Calcd for C_22_H_27_F_3_N_2_: C, 70.19; H, 7.22; N, 7.44. Found: C, 70.00; H, 7.19; N, 7.40.

#### 2-(4-Substituted-phenyl)sparteines (7). (General method)

A solution of the suitable 2-dehydrosparteine derivative (1.6 mmol) in 20 mL of EtOH was hydrogenated at r.t. and atmospheric pressure in the presence of 10% Pd/C (0.1 g). After about 1 h, the calculated volume of H_2_ was absorbed. The catalyst was removed and the solvent was evaporated *in vacuo*, affording a residue that was crystallized from the indicated solvent.

##### 2-Phenylsparteine (7a)

Yield: 62%. Mp 91–92 °C (Et_2_O) [lit.16a: oil, b.p. 160–161 °C, high *vacuum*]. ^1^H NMR (200 MHz, CDCl_3_): 1.04–2.18 (m, 20 H), 2.37–3.08 (m, 5 H), 7.19–7.55 (m, 5 H, ArH). Anal. Calcd for C_21_H_30_N_2_: C, 81.24; H, 9.74; N, 9.02. Found: C, 81.39; H, 9.84; N, 9.08.

##### 2-(4’-Fluorophenyl)sparteine (7b)

Yield: 70%. Mp 101–102 °C (Et_2_O). ^1^H NMR (200 MHz, CDCl_3_): 1.02–2.16 (m, 21 H), 2.22–3.05 (m, 4 H), 6.90–7.12 (m, 2 H, ArH), 7.23–7.48 (m, 2 H, ArH). ^13^C NMR (50 MHz, CDCl_3_): 162.96, 158.11, 140.63, 127.74, 67.46, 65.28, 63.53, 57.44, 54.70, 52.69, 36.08, 35.11, 33.48, 32.76, 28.85, 26.80, 24.87, 23.90, 23.48. Anal. Calcd for C_21_H_29_FN_2_: C, 76.90; H, 8.90; N, 8.53. Found: C, 76.79; H, 8.97; N, 8.50.

##### 2-(4’-Methoxyphenyl)sparteine (7d)

Yield: 72%. Oil. [lit.16b: oil, b.p. 188 °C, high *vacuum*]. ^1^H NMR (200 MHz, CDCl_3_): 1.03–2.10 (m, 20 H), 2.35–2.57 (m, 2H), 2.69–2.93 (m, 3 H), 3.82 (s, OCH_3_), 6.85 (d, *J* = 10.0 Hz, 2 H, ArH), 7.24 (d, *J* = 10.0 Hz, 2 H, ArH). Anal. Calcd for C_22_H_32_N_2_O: C, 77.60; H, 9.47; N, 8.23. Found: C, 77.28; H, 9.45; N, 8.07. Dihydrochloride Mp 245–246 °C. Anal. Calcd for C_22_H_34_Cl_2_N_2_O + H_2_O: C, 61.24; H, 8.41; N, 6.49. Found: C, 61.68; H, 8.43; N, 6.16.

##### 2-(4’-Tolyl)sparteine (7e)

Yield: 40%. Mp 80–82 °C (Et_2_O) [lit.^24^: 81–83 °C]. ^1^H NMR (200 MHz, CDCl_3_): 1.04–2.18 (m, 20 H), 2.25–2.60 (m, 2H and 2.33, s, CH_3_, superimposed), 2.70–2.96 (m, 3 H), 7.12 (d, *J* = 10.0 Hz, 2 H, ArH), 7.23 (d, *J* = 10.0 Hz, 2 H, ArH). ^13^C NMR (50 MHz, CDCl_3_): 142.02, 134.98, 127.96, 126.28, 68.00, 65.34, 63.46, 57.50, 54.70, 52.73, 36.08, 35.15, 33.50, 32.83, 28.94, 26.86, 24.91, 23.90, 23.62, 20.07. Anal. Calcd for C_22_H_32_N_2_: C, 81.43; H, 9.94; N, 8.63. Found: C, 81.47; H, 10.05; N, 8.67.

##### 2-(4’-Trifluoromethyl)sparteine (7f)

Yield: 48%. Mp 68–70 °C (acetone). ^1^H NMR (200 MHz, CDCl_3_): 1.03–2.14 (m, 20 H), 2.30–2.57 (m, 2H), 2.70–3.07 (m, 3 H), 7.43 (d, *J* = 10.0 Hz, 2 H, ArH), 7.66 (d, *J* = 10.0 Hz, 2 H, ArH). ^13^C NMR (50 MHz, CDCl_3_): 146.90, 142.97, 127.52, 126.61, 123.89, 67.95, 65.13, 63.61, 57.62, 54.69, 52.55, 35.92, 35.04, 33.33, 32.66, 28.75, 26.65, 24.76, 23.80, 23.40. Calcd for C_22_H_29_F_3_N_2_: C, 69.82; H, 7.72; N, 7.40. Found: C, 69.62; H, 7.80; N, 7.32.

##### 2-(4’-Chlorophenyl)sparteine (7c)

A solution of 2-(4’-chlorophenyl)-2-dehydrosparteine (0.73 mmol, 0.25 g, **6c**) in 4 mL of EtOH was added with NaBH_4_ (3.3 mmol, 125 mg) and refluxed for 4 h with stirring. After removing the solvent, the residue was treated dropwise, and in the order, with 3 mL of H_2_O, 3 mL of 2N NaOH and extracted with Et_2_O. The organic layer was dried (Na_2_SO_4_), filtered, and concentrated to dryness, affording the title compound.

Yield: 95%. Mp 75–77 °C (Et_2_O). ^1^H NMR (200 MHz, CDCl_3_): 1.00–3.10 (m, 23 H), 3.38–3.57 (m, 2 H), 7.15–7.38 (m, 4 H, ArH). ^13^C NMR (50 MHz, CDCl_3_): 143.53, 130.92, 127.71, 127.43, 67.57, 65.21, 63.54, 57.53, 54.70, 52.68, 35.96, 35.10, 33.48, 32.74, 28.81, 26.79, 24.86, 23.88, 23.45. Anal. Calcd for C_21_H_29_ClN_2_: C, 73.12; H, 8.47; N, 8.12. Found: C, 72.55; H, 8.49; N, 7.93.

### Docking studies

All the compounds were built, parameterized (Gasteiger–Huckel method) and energy minimized within MOE using MMFF94 forcefield^25^. For the newly synthesized sparteine derivative **7b–f** the two *R* and *S* enantiomers were taken into account and built *in silico*.

Docking calculations within the X-ray structure of human sigma-1 receptor (pdb code = 5HK1) were performed using the MOE-DOCK tool, implemented in MOE. The compound best-docked pose, evaluated in terms of “London dG”, was refined by energy minimization (MMFF94) and rescored according to “Affinity dG” (kcal/mol of total estimated binding energy). The final score (based on the latest refinement step which has been applied) namely S score, was considered to prioritize any ligand conformation.

In addition, the best docking geometry was refined by ligand/protein complex energy minimization (CHARMM27) and successfully assessed using a short ∼1 ps run of molecular dynamics (MD) at constant temperature, followed by an all-atom energy minimization (LowModeMD implemented in MOE software). This kind of module was allowed to perform an exhaustive conformational analysis of the ligand–receptor binding site complex, as we already discussed about other case studies, where it proved to be useful for a preliminary evaluation of docking poses^26^.

### Pharmacological studies

#### Animals

Dunkin–Hurtley male guinea pigs (300–500 g), obtained from Harlan Italy (S. Pietro al Natisone, Italy), were kept in controlled environmental conditions (temperature: 23 ± 2 °C; light–dark cycle: 7 a.m. to 7 p.m.). Animals had free access to a standard laboratory diet and to water. All animal-use procedures described in this paper were in accordance with the National Institutes of Health guidelines for the Care and Use of Laboratory Animals and comply with the ethical principles and guidelines adopted by the European Community, law 86/609/CEE. The experimental protocol was approved by the local veterinary committee.

In order to obtain myocardial tissues depleted in endogenous catecholamines, the animals were treated daily for 2 d with reserpine (2 mg/kg i.p.) and anesthetized with methoxyflurane before sacrifice.

#### Assessment of inotropic and chronotropic activities on isolated atrial preparations

The atria were separated from ventricles and suspended vertically in a 30 mL organ bath containing a physiological salt solution constantly gassed by 95% O_2_ and 5% CO_2_, at 30 °C. The bathing solution contained (mM): NaCl 120, KCl 2.7, CaCl_2_ 1.36, MgCl_2_ 0.09, NaH_2_PO_4_ 0.4, NaHCO_3_ 12 and glucose 5.5 (pH = 7.5). The resting tension was adjusted at 10.0 mN and the developed tension was recorded isometrically by means of a high-sensitivity transducer (Ugo Basile, type DYO Comerio, Italy) connected to a PC-based Acqknowledge acquisition system (BIOPAC Systems, Inc., 42 Aero Camino Santa Barbara, CA). The drugs were added to the perfusion fluid after 90 min of equilibration. Since the atria were isolated from reserpine treated animals, before the beginning of experiments, the depletion of catecholamines was verified by lack of any positive inotropic effect induced by tyramine (2 µg/mL). Experiments were performed only in preparations that did not respond to tyramine. Test compounds and milrinone were added cumulatively and the effects caused by each drug concentration were recorded up to the maximum response before a higher concentration was added. The effects of each compound on the force of contraction and the frequency were expressed as the percent increase/decrease over controls (Δ%). Compounds were dissolved in physiological solution or in the stoichiometric 0.5 N HCl, while milrinone was dissolved in dimethyl sulfoxide (DMSO). The final concentration of DMSO in the medium did not influence the basal activity of the atrial preparations. The statistical comparisons between treatment and control data were performed by ANOVA followed by Bonferroni *t* test; a value of *p* < 0.05 was considered statistically significant.

#### General pharmacological screening

For *in vivo* tests, compounds were generally administered orally (*p.o*) by gavage; they were prepared as aqueous solutions or finely homogenized suspensions in "tween 80" (2%). In a few cases, the substances were introduced intraperitoneally (*i.p.*). Groups of three or five animals (mice or rats) were used. For *in vitro* assays, sometimes it was necessary to increase the solubility by means of DMSO in a concentration not interfering with the tests (0.1% for platelet aggregation and 0.5% for all the others).

Doses (mg/kg) or concentrations (µg/mL) indicated in the following methods were the highest commonly utilized; when significant activity was detected, lower doses or concentrations were tested in order to define the minimal effective ones.

#### Maximal tolerated dose, autonomic signs, and Irwin test

Three mice were dosed at 300 mg/kg *p.o.* and 100 mg/kg *i.p.* for observation of acute toxic symptoms or autonomic effects during the subsequent 72 h. If none was noted, pharmacological evaluation proceeded employing doses and concentrations for each test based on appropriate multiple of doses required by suitable reference compounds. If acute toxicity was observed initially, the maximal tolerated dose was determined and pharmacological screening doses were reduced proportionally.

Before and 1 h after dosing mice with test samples, 10 parameters indicating motor stimulation (irritability, hyperactivity, increased palpebral size, increased startle response, increased response to touch, increased exploration, piloerection, strand tail, tremors, convulsions) were measured. Normalcy for each is 0; maximum abnormal condition for each score 2 × 10 × 3 mice = 60. Scores greater than 12 denote significant stimulation. Similarly, 10 parameters (pinna reflex, spontaneous activity, palpebral size, startle response, touch response, reactivity, placing, righting reflex, exploration, and ataxia) were measured for behavioral depression. Each parameter scores 2 points for normalcy for a total of 2 × 10 × 3 = 60 points possible. Scores below 40 denote significant behavioral depression.

#### Blood pressure

Two spontaneously hypertensive rats (SHR) with systolic blood pressures ranging between 180 and 220 mmHg were used. Blood pressure was determined by tail-cuff method in a temperature-controlled environment (32 ± 1 °C) before, 1, 2, and 4 h after test substance administration *p.o*. (100 mg/kg). Reduction in systolic blood pressure by more than 10% at any two of the aforementioned three consecutive time intervals is considered significant.

#### Heart rate

The same SHRs employed in the preceding test were used. Heart rate was recorded by ECG, immediately after blood pressure recordings, before and 1, 2, and 4 h post-treatment *p.o*. (100 mg/kg). An increase or a decrease in heart rate greater than 20% from pretreatment control readings indicates significant tachycardia or bradycardia, respectively.

#### Antiarrhythmic activity

The substance was administered *i.p*. (30 mg/kg) to a group of 3 mice 30 min before exposure to deep chloroform anesthesia and observed during the following 15 min period. The absence of ECG recorded cardiac arrhythmias and heart rate above 200 beats/min (usually 400–480 beats/min) in at least two mice indicates significant protection.

#### *In vitro* inotropic effect

Test substance (10 µg/mL)-induced variation in contractile force of electrically stimulated (95% of maximum, 150 beats/min) guinea pig left atria, bathed in physiological salt solution containing one third normal calcium concentration at 32 °C, by more than 40% indicates significant (±) inotropic activity. Only inamrinone sensitive preparations were used.

#### *In vitro* chronotropic effect

Test substance (10 µg/mL)-induced change in atrial rate in spontaneously beating guinea pig atria, bathed in physiological salt solution at 37 °C, by more than 10% is considered significant.

#### Effects on diuresis, saluresis, and kaluresis

Groups of three overnight-fasted rats were used. Each group was hydrated with distilled water (25 mL/kg, *p.o.*) administered together with test substance (30 mg/kg, *p.o.*) or vehicle. Urine volume was measured over the ensuing 6 h period and analyzed for Na^+ ^ and K^+ ^contents, expressed as µeq/100 g body weight. A greater than 50% increase or decrease in urine volume in test versus control animals was considered a significant effect. A greater than two-fold increase of Na^+ ^ and K^+ ^excretion in test versus control animals was considered a significant saluretic or kaluretic effect.

#### *In vitro* tracheal relaxation

The strip preparations of isolated guinea pig trachea was used to study the contractile tension, placed in physiological salt solution at 37 °C under 4.9 mN. Test substance (100 µg/mL) inhibition of tracheal tone by more than 50%, relative maximal relaxation induced by 0.3 µg/mL epinephrine, indicates significant activity. If test substance-induced relaxation is antagonized more than 50% by propranolol (2 µg/mL), a well known β_2_-adrenoceptor blocker, an agonist action is indicated.

#### Anti-inflammatory activity

In a group of three overnight-fasted rats, test substance was administered *p.o*. (100 mg/kg) 1 h before right hind paw intra-plantar injection of carrageenan (0.1 mL, 1% suspension). Inhibition of paw edema by more than 30% 3 h after carrageenan administration indicates acute anti-inflammatory activity.

#### Analgesic activity

*Writhing test:* Test sample was administered *p.o*. (100 mg/kg) to a group of 3 mice 1 h before injection of phenylquinone (2 mg/kg, *i.p.*), greater than 50% inhibition of the number of twists per group of animals observed during 5–10 min after phenylquinone, relative to a vehicle-treated group, indicates possible analgesic activity.

*Formalin test:* Test sample was administered *p.o*. (30 mg/kg) to a group of 5 mice 1 h before sub-plantar injection of formalin (0.02 mL, 1% sol.) into the right hind paw. Reduction of the induced paw licking time recorded during the following 20–30 min period by more than 50% indicates analgesic activity.

#### *In vitro* platelet aggregation inhibition

*Sodium arachidonate induced aggregation:* Test substance (10 µg/mL) inhibition by more than 50% of maximum non-reversible platelet aggregation (rabbit platelet-rich plasma, RPP) induced by sodium arachidonate (50 µg/mL) indicates significant activity.

*ADP induced aggregation:* Test substance (100 µg/mL) inhibition by more than 50% of maximum non-reversible platelet aggregation (RPP) induced by adenosine diphosphate (ADP, 0.4–0.8 µg/mL) indicates significant activity.

*PAF induced aggregation:* Test substance (10 µg/mL) inhibition by more than 50% of maximum non-reversible platelet aggregation (RPP) induced by platelet activating factor (PAF-acether, 10–20 ng/mL) indicates significant activity.

## Results and discussion

### Synthesis

The required compounds were prepared by reacting (±)-lupanine (extracted from seeds of *Lupinus albus*) with the proper 4-substituted phenylmagnesium bromides.

The initially formed carbinolamines could not be isolated even in the mildest working up procedures. The reduction of 2-dehydrosparteine derivatives (6) was commonly performed by catalytic hydrogenation (Pd/C), or by the action of sodium borohydride in the case of compound **6c**, in order to avoid the chlorine hydrogenolysis. The methoxy derivative **6d** was rather unstable and was immediately converted into the saturated compound **7d**.

### Biological results

#### Pharmacology: general considerations

Ten compounds (**6b**, **6c**, **6e**, **6f**, and **7a–f**) were tested *in vitro* for inotropic and chronotropic activities on spontaneously beating atria obtained from guinea pigs pretreated with reserpine in order to avoid the influence of endogenous catecholamines released from nerve terminals (Tables 1 and 2). Two compounds could not be tested either for shortage of sample (**6a**) or for its chemical instability (**6d**).

**Scheme 1:**
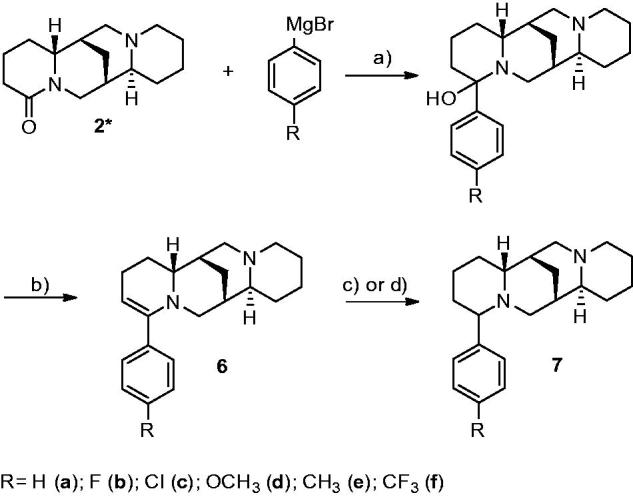
Reagents and conditions: (a) dry Et_2_O; reflux, 2 h; (b) dil. HCl; (c) H_2_/Pd-C, EtOH, r.t.; (d) NaBH_4_, EtOH; reflux, 4 h. *(±)-lupanine, for the sake of simplicity, only one enantiomer is shown.

**Table 1.** Effect of compounds **6b**, **c**, **e**, **f**, **7a**–**f**, and milrinone (**M**) on the force of contraction (inotropism) of isolated atria of guinea pigs pretreated with reserpine.

Developed tension (Δ%)									
Comp.	R=	10^−7^ M	3 × 10^−7^ M	10^−6^ M	3 × 10^−6^ M	10^−5^ M	3 × 10^−5^ M	10^−4^ M	3 × 10^−4^ M
**6b**^a^	F	1.82 ± 0.02	3.01 ± 0.03	7.07 ± 0.04	15.88 ± 0.07	29.44 ± 0.11	32.88 ± 0.14	−28.57 ± 0.12	−57.14 ± 0.22
**6c**^a^	Cl	3.70 ± 0.13	7.40 ± 0.11	3.70 ± 0.09	−3.70 ± 0.08	−14.80 ± 0.12	−29.60 ± 0.14	tox	tox
**6e**^a^	CH_3_	4.10 ± 0.01	10.64 ± 0.05	19.38 ± 0.07	28.44 ± 0.12	26.99 ± 0.17	24.69 ± 0.16	−29.76 ± 0.13	−58.33 ± 0.24
**6f**^a^	CF_3_	0.65 ± 0.01	0.78 ± 0.02	0.09 ± 0.01	−9.02 ± 0.02	−12.93 ± 0.04	−33.09 ± 0.15	−48.21 ± 0.57	−74.10 ± 0.86
**7a**^b^	H	0.00 ± 0.00	0.00 ± 0.00	1.56 ± 0.09	2.75 ± 0.10	0.72 ± 0.03	−3.73 ± 0.08	tox	tox
**7b**^a^	F	1.50 ± 0.23	2.14 ± 0.18	5.17 ± 0.24	9.53 ± 0.24	20.85 ± 0.36	29.99 ± 0.37	tox	tox
**7c**^b^	Cl	0.00 ± 0.00	0.00 ± 0.00	−4.17 ± 0.24	−16.67 ± 0.18	−29.17 ± 0.20	tox	tox	tox
**7d**^b^	OCH_3_	0.01 ± 0.01	0.34 ± 0.02	0.83 ± 0.03	1.65 ± 0.04	1.39 ± 0.04	−5.01 ± 0.07	tox	tox
**7e**^b^	CH_3_	1.97 ± 0.15	2.55 ± 0.17	3.27 ± 0.09	9.76 ± 0.10	38.81 ± 0.35	68.96 ± 1.15	110.71 ± 2.77	116.50 ± 3.41
**7f**^b^	CF_3_	1.64 ± 0.16	8.63 ± 0.21	17.45 ± 0.17	27.02 ± 0.21	43.08 ± 0.40	41.32 ± 0.25	tox	tox
**M**^c^		nt	nt	16.69 ± 0.95	30.24 ± 0.75	38.68 ± 1.25	46.16 ± 0.80	52.70 ± 0.83	52.25 ± 1.13

Test compounds were added cumulatively to the bathing fluid, and inotropic effect was recorded for 5 min after they reached maxima, before adding a higher concentration. The value of basal force of contraction was 7.23 ± 0.32 mN. Results are means ± SEM of 6–10 experiments. Negative value indicates negative inotropism (decrease of developed tension). nt: not tested; tox: toxicity.

a Dissolved in 0.5N HCl.

b Dissolved in physiological solution.

c Dissolved in DMSO.

**Table 2.** Effect of compounds **6b**, **c**, **e**, **f**, **7a**–**f,** and milrinone (**M**) on the frequency rate (chronotropism) of isolated atria of guinea pigs pretreated with reserpine.

Frequency (Δ%)									
Comp.	R	10^−7^ M	3 × 10^−7^ M	10^−6^ M	3 × 10^−6^ M	10^−5^ M	3 × 10^−5^ M	10^−4^ M	3 × 10^−4^ M
**6b**^a^	F	−1.33 ± 0.19	−1.77 ± 0.18	−3.35 ± 0.21	1.56 ± 0.18	0.73 ± 0.09	0.55 ± 0.04	−2.50 ± 0.18	−5.00 ± 0.22
**6c**^a^	Cl	0.00 ± 0.00	0.00 ± 0.00	0.00 ± 0.00	−4.00 ± 0.12	−28.00 ± 0.12	−32.00 ± 0.43	tox	tox
**6e**^a^	CH_3_	1.59 ± 0.23	3.09 ± 0.25	5.79 ± 0.31	5.97 ± 0.32	3.11 ± 0.27	2.02 ± 0.24	−7.25 ± 0.26	−12.05 ± 0.29
**6f**^a^	CF_3_	−0.45 ± 0.20	−0.77 ± 0.21	−1.94 ± 0.15	−3.83 ± 0.18	−7.74 ± 0.64	−17.96 ± 0.22	−20.83 ± 0.84	−40.97 ± 0.71
**7a**^b^	H	0.45 ± 0.03	0.98 ± 0.07	1.31 ± 0.09	1.50 ± 0.07	2.03 ± 0.11	−4.25 ± 0.21	tox	tox
**7b**^a^	F	0.28 ± 0.03	0.67 ± 0.04	0.78 ± 0.05	1.33 ± 0.04	5.21 ± 0.21	4.41 ± 0.31	tox	tox
**7c**^b^	Cl	3.70 ± 0.19	4.35 ± 0.21	4.35 ± 0.18	4.35 ± 0.21	−13.04 ± 0.31	−26.08 ± 0.41	tox	tox
**7d**^b^	OCH_3_	1.14 ± 0.18	2.39 ± 0.19	6.84 ± 0.30	6.99 ± 0.30	10.96 ± 0.41	5.50 ± 0.39	tox	tox
**7e**^b^	CH_3_	0.71 ± 0.03	0.92 ± 0.07	1.12 ± 0.10	2.27 ± 0.12	7.39 ± 0.20	19.33 ± 0.28	25.73 ± 0.31	27.27 ± 0.32
**7f**^b^	CF_3_	0.58 ± 0.02	0.58 ± 0.03	5.69 ± 0.24	10.88 ± 0.48	14.39 ± 0.38	19.22 ± 0.41	tox	tox
**M**^c^		nt	nt	3.01 ± 0.24	9.12 ± 0.05	11.52 ± 0.33	18.22 ± 0.84	24.46 ± 0.75	32.81 ± 1.04

Test compounds were added cumulatively to the bathing fluid, and chronotropic effect was recorded for 5 min after they reached maxima, before adding a higher concentration. The value of basal heart rate was 132.0 ± 3.5 bpm. Results are means ± SEM of 6–10 experiments. Negative value indicates a negative chronotropic effect. nt: not tested; tox: toxicity.

a Dissolved in 0.5N HCl.

b Dissolved in physiological solution.

c Dissolved in DMSO.

Moreover, the so far unpublished results of the previous pharmacological screening of compound **7b**, in comparison with milrinone, were included, providing some additional information on other cardiovascular and related activities and on toxicity of this kind of compounds (Table 3).

**Table 3.** General pharmacological screening of compound **7b** and milrinone (**M**).

Test	Dose (mg/kg) or Conc. (μg/mL)	Considered parameter	Significant criterion	7b	M	Additional reference drug^a^
MTD and Irwin test^b^	*p.o.* 300	Number animals (died/treated)		3/3	nt	
	100			3/3	3/3 (48h)	
	30			0/3	3/3 (48h)	
	*i.p.* 100			3/3	3/3	
	50			nt	3/3 (24h)	
	30			0/3	3/3 (48h)	
Blood pressure	*p.o.* 30	Δ% after 1 and 4 h	>10%	−4/0	nt	
	10				−13/13	
	5				−3/−3	
Heart rate	*p.o.* 30	Δ% after 1 and 4 h	>20%	+3/+5	+7/+8	
CHCl_3_ arrhythmia	*i.p.* 30	N° protected animals/treated		1/3		Lidocaine: 3/3
	5		>1/3		0/3	Quinidine (100 mg/kg): 3/3
Inotropism^c^ (*in vitro*)	10	Δ%	>40%	+56	+72	Trequinsin: +48
	3			+31	+60	
	0.5				+45	
Chronotropism (*in vitro*)	10	Δ%	>10	−23	nt	Quinidine: −20; mecamylamine: −14
	5				+11	
	3			−10		
	2.5				+7	
	1			−3		
Saluresis	*p.o.* 30	Urine Na^+^ μeq treated/control	>2	4.5		Furosemide (5 mg/kg): 3.0 Triamterene (2.5 mg/kg): 2.6
	10			4.1	0.5	
	3			3.4		
Diuresis	*p.o.* 30	Urine vol. treated/control	>1.5	2.2	nt	Amiloride (20 mg/kg): 1.6 Hydroflumethiazide (2.5 mg/kg): 1.7
	10			2.0	0.4	
	3			1.8		
Tracheal contractile tension (*in vitro*)	100	Relaxation (%)	>50%	nt	100	
	30			16	nt	Theophylline: 60
	1.0			nt	50	
	0.5			nt	23	
Anti-inflammatory (paw edema)	*p.o.* 100	Inhibition (%)	>30%	42	nt	Aspirin (150 mg/kg): 40 Phenylbutazone (50 mg/kg): 33 Hydrocortisone (25 mg/kg): 32
	30			34	nt	
	10			0	20	
Phenylquinone writhing	*p.o.* 300	Writhing inhibition (%)	>50%	nt	42	Aspirin (50 mg/kg): 68
	30			60	nt	
	10			20	nt	
Formalin algesia	*p.o.* 30	Licking time reduction (%)	>50%	89	nt^d^	
	10			27		

nt: not tested.

a When not otherwise specified, the drug reference dosage corresponds to that of column 2.

b MTD: maximal tolerated dose; neither significant motor stimulation nor behavioral depression were observed.

c Not blocked by propranolol.

d Only compounds active in the writhing test were assayed.

#### Inotropic and chronotropic activities

As it is illustrated in Table 1, the dehydrosparteine derivatives **6b** and **6e** showed a concentration-dependent positive inotropic effect that at the higher concentrations of 100 and 300 µM was converted to negative inotropism. A similar positive inotropism was observed for compounds **7b** and **7f** which, however, at 100 and 300 µM induced overt toxicity characterized by the appearance of arrhythmias and functional derangement leading to the irreversible stop of atrial contractions. It is worth noting that, at the highest non-toxic concentration (30 µM), compound **7b** displayed an inotropic effect (+30%) comparable with that previously observed on electrically driven atria of non-reserpinized guinea pig (+56%, see Table 3).

At concentrations up to 30 µM, compounds **6b**, **6e**, and **7b** exhibited only modest (<6%) positive or negative chronotropic activities (Table 2), while compound **7f** exhibited moderate positive chronotropism (up to +19%). At higher concentrations, all the cited compounds showed either negative chronotropism or toxicity (Table 2).

Very interestingly, the 2-(4-tolyl)sparteine **7e** exhibited a steep enhancement of contractile strength up to the maximal concentration tested (300 µM), with an *E*_max_ of 116.5 ± 3.4% of the basal contractile force. This powerful positive inotropism was associated with only a moderate increase of the beating rate that reached +27.3 ± 0.3% at the highest concentration (300 µM).

The remaining compounds, including 2-phenylsparteine **7a** and 2-(4-methoxyphenyl)sparteine **7d** (tested in the past^16^^,^^17^ for antifibrillatory activity), were endowed with modest positive inotropism at the lower concentrations, followed by increasing negative inotropism or toxicity at the higher concentrations. Similar effects were observed concerning the beating frequency.

Further, the inotropic activity of **7e** was compared with that of two well-known inotropic drugs digoxin and milrinone. Figure 2 reports the concentration–effect curves of the three inotropic agents showing that digoxin was the most potent, increasing the basal contractile force by 50% at 0.9 µM concentration, while compound **7e** was the most efficacious with an *E*_max_ = 116% at 300 µM concentration. Milrinone was moderately potent but the least active. Concentrations of digoxin higher than 3 µM could not be tested because of toxicity.

**Figure 3.**
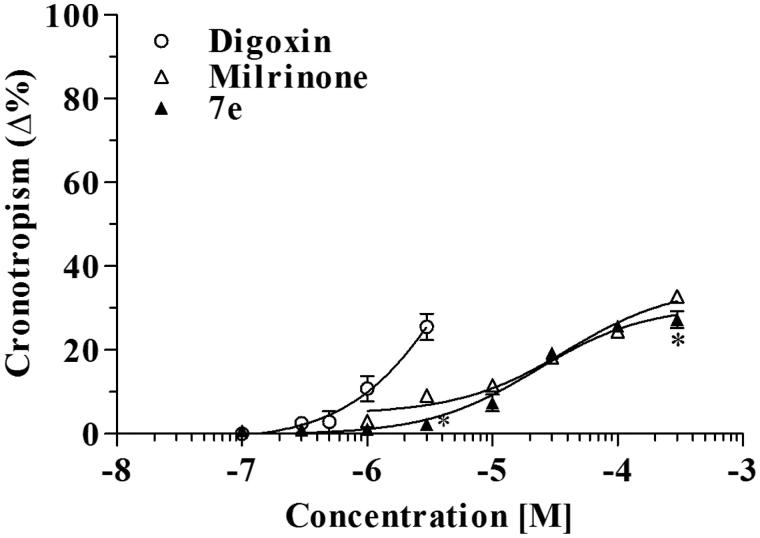
Chronotropic effects of digoxin, milrinone and compound **7e**. *p < 0.05 compound **7e** versus milrinone.

Comparing chronotropism of the three agents, it was observed (Figure 3) that digoxin increased the frequency up to 25% on basal value at the concentration of 3 µM; furthermore, higher concentrations induced high toxicity with arrhythmias and atrial block. Instead, the compound **7e** slightly increased frequency, much less than digoxin, and also less than milrinone without ever causing severe cardiac toxicity (Figure 3).

The structural features that characterized the studied compounds (the presence or the absence of the enaminic double bond and consequent variation of the configuration of the tetracyclic system; nature of the substituent on the aromatic moiety) clearly influence both inotropic and chronotropic activities. On the whole, the 2-dehydrosparteine derivatives were more prone to produce negative inotropism and, even more, negative chronotropism, in comparison with the saturated compounds. However, the influence on the activity exerted by the presence of the enaminic double bond is not constant in the four couples of compounds bearing the same substituent on the aromatic moiety (compare **6b**/**7b**; **6c**/**7c**; **6e**/**7e**; **6f**/**7f**). The influence on the activity of the structural features is also variable in relation to the tested concentrations, because they may exert different effects in relation to permeability of cardiac tissue and intracellular targets responsible for the force and the frequency of cardiac contraction.

Anyhow, among the 2-dehydrosparteines (**6**), the highest and lowest inotropic activities were found for the fluorophenyl (**6b**) and the trifluoromethylphenyl (**6f**) derivatives, respectively. On the contrary, among the saturated compounds (**7**), the fluorophenyl (**7b**) was less active than the trifluoromethylphenyl (**7f**) derivative that was the most active of the whole set of compounds up to the 10 µM concentration, beyond which it was largely surmounted by the methylphenyl analog **7e**. Indeed, the concentration–effect curve of **7f** is rather flattened and parallel to that of milrinone up to 30 µM (Table 1), while that of compound **7e** is characterized by its steepness, paralleling (at the higher concentrations) that of digoxin (Figure 2).

#### Pharmacological screening of compound **7b**and milrinone

The most interesting results of the general pharmacological screening of compounds **7b** in comparison with milrinone are illustrated in Table 3.

First of all, it is observed that the sparteine derivative was moderately toxic, but less than milrinone. Indeed, in both cases at the dose of 300 mg/kg *p.o.* and 100 mg/kg *i.p.* all treated mice died, but at lower dose (30 mg/kg *p.o.* and *i.p.*), no death was observed with the sparteine derivative within the 72 h of observation, whereas with milrinone all animals died within 48 h (Table 3). For comparison, in guinea pig, sparteine sulfate exhibited an *i.p.* MLD between 42 and 55 mg/kg^27^, and digoxin exhibited an oral DL_50_ = 3.5 mg/kg. In the Irwin test, compound **7b** and milrinone showed neither significant motor stimulation nor behavioral depression.

Concerning the cardiovascular system, it is observed that the 2-(4-fluorophenyl)sparteine **7b** did not exhibit any activity on the blood pressure and the heart rate in spontaneously hypertensive rats, while milrinone showed a moderate reduction of pressure. Somewhat surprising, **7b** did not display significant antiarrhythmic activity in the chloroform induced arrhythmia assay. In previous studies^16^^,^^17^, the analogous compounds **6a**, **6d**, **7a**, and **7d**, as well as sparteine, were found to display antifibrillatory activity on isolated frog heart.

More importantly, a net positive inotropic activity was found for compound **7b** and milrinone on isolated, electrically driven guinea pig left atria. Negative chronotropic action (comparable to that of quinidine) was observed for the sparteine derivative on spontaneously beating guinea pig right atria, while milrinone displayed positive activity on beating frequency. While the results reported in Tables 1 and 2 were obtained from reserpinized guinea pig atria, those reported in Table 3 were from untreated tissue and the endogenous amines can affect the observed ino- and chronotropic activities of **7b** and milrinone.

**Figure 4.**
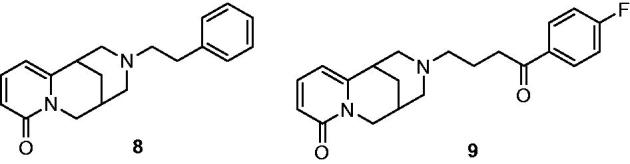
Inotropic cytisine derivatives.

The positive inotropism of compound **7b** is related neither to ß-adrenergic receptor activation, since it was not blocked by propranolol nor to phosphodiesterase inhibition, since the reduction of tone in guinea pig tracheal strips was not observed, even at 30 µg/mL concentration, while milrinone clearly reduced the tone of trachea still at 1 µg/mL (−50%). High positive inotropic activity on electrically driven guinea pig left atria (from non-reserpinized animal) was observed^19^ for a couple of cytisine derivatives (**8** and **9**; Figure 4). Also in this case, the inotropic activity was not blocked by propranolol, and no reduction of spontaneous tone of trachea strips was observed.

Compound **7b** exhibited potent saluretic and diuretic activities (Table 3) that were quite higher than those expressed by furosemide, triamterene, amiloride, and hydroflumethiazide. These activities were not shared by milrinone.

The combination of diuretic activity with positive inotropism may result very valuable to relieve symptoms of CHF; therefore, the presence of diuretic and saluretic activities should be investigated for the whole set of sparteine derivatives.

Finally, **7b** exhibited moderate analgesic (writhing test and formalin algesia) and anti-inflammatory (carragenin induced edema) activities that were not shared by milrinone. The anti-inflammatory activity seems unrelated to Cox inhibition, since inhibition of arachidonic acid (AA) induced platelet aggregation was not observed; milrinone completely inhibited the AA induced aggregation even at 0.1 µg/mL concentration.

**Figure 5.**
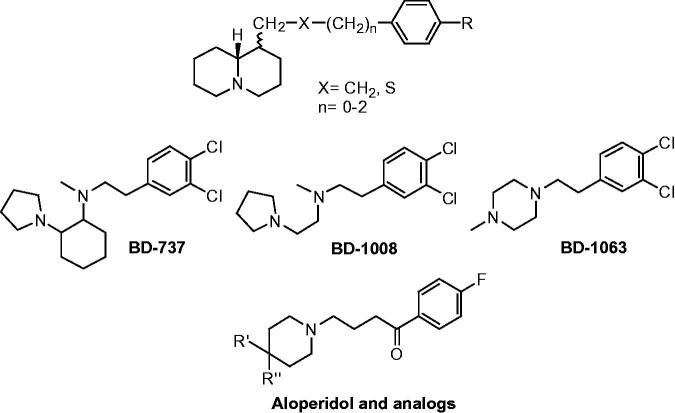
Some arylalkyl amines with high affinity for sigma-1 receptor.

Thus, the combination of polycyclic alkaloidal frameworks with particular aromatic moieties, as in compounds **6–9**, seems suitable to generate positive inotropism through a mechanism not related to adrenergic stimulation or PDE inhibition. The common structural feature of compounds **6–9** resembles that of some arylalkyl quinolizidines^28^ and some arylalkyl amines, like BD-737, BD-1008, BD-1063, and the butyrophenone antipsychotics (Figure 5) that are characterized by high affinity to sigma-1 receptor^29^.

Sigma receptor ligands present well-documented effects on cardiac muscle^30–34^, thus the possibility that the inotropic activity of compounds **6–9** is chaperoned by interaction with sigma receptor is tentatively advanced. This hypothesis is supported by the excellent fitting of molecules **7b** and **7e** on the binding site of sigma 1 receptorial protein (see further). Anyhow, further investigations are needed to define the mechanism of the inotropic activity of the relevant sparteine derivatives.

#### Docking studies

Sigma ligands are characterized by a rather large variety of structures and up to now, the development of potent sigma-1 ligands was efficiently driven by computational methods, based on homology studies of the target^35^ and also fulfilling a pattern of pharmacophore features exhibited by several series of derivatives, as discussed in the literature^36^. Indeed, most of sigma-1 ligands were characterized by the positive ionizable group and hydrogen bond acceptor functions connecting at least two hydrophobic cores. Thus, for sigma-1 ligands, a recurrent binding mode was proposed in literature, suggesting key contacts with an aspartic acid residue (D126), as confirmed by mutagenesis experiments^37^.

More recently, the X-ray crystallographic structure of the human sigma-1 receptor became available, definitively providing more reliable avenues to pave the way for the rational design of further molecules (pdb code = 5HK1; resolution = 2.51 Å)^38^.

This computational work was aimed to explore the reliability of the sigma-1 protein as putative biological target to be proposed for the newly derivatives here discussed. The main issues to be addressed were to clarify, through docking studied of the two series of compounds **6–7**, the role played by the sparteine nucleus and by the phenyl ring with respect to the co-crystallized ligand PD144418, bearing a 1-propyl-3-[3-(*p*-tolyl)isoxazol-5-yl]-1,2,5,6-tetrahydropyridine structure (Table 4).

**Table 4.** Final score (S) derived from MOE docking calculations

Compound	S Score (kcal/mol)
**6b**	−96.312
**6c**	−91.364
**6e**	−95.678
**6f**	−98.364
**7a**	−121.543
**7b**	−112.003
**7c**	−114.301
**7d**	−112.378
**7e**	−123.137
**7f**	−121.312
**PD144418**	−132.632
sparteine	−88.721

**Figure 6.**
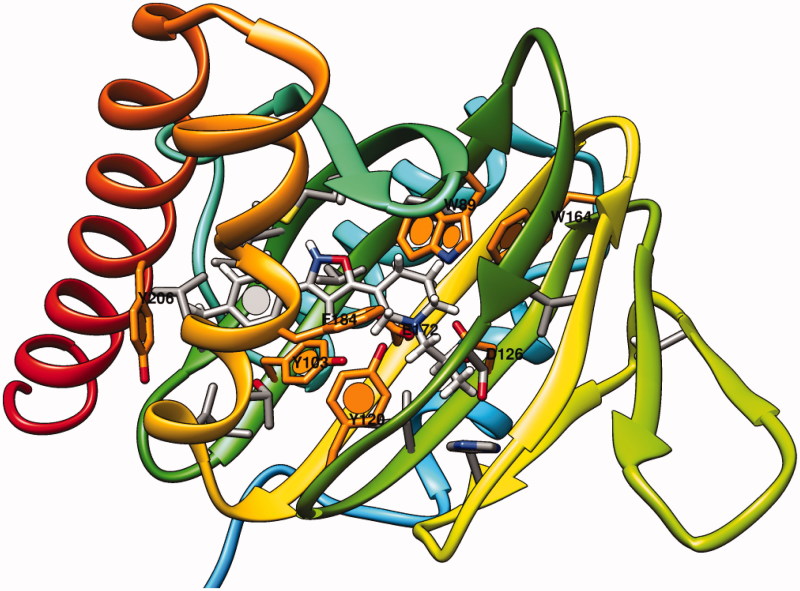
X-ray crystallographic data of the human sigma-1 receptor and of the ligand PD144418. The most important residues are labeled and colored in orange.

As shown in Figure 6, PD144418 was engaged in salt bridges involving the protonated nitrogen atom of the tetrahydropyridine group and D126, E172, giving a good validation of the aforementioned homology models and mutagenesis data. On the contrary, the propyl chain and the phenyl ring detected hydrophobic contacts with the surrounding amino acids.

**Figure 7.**
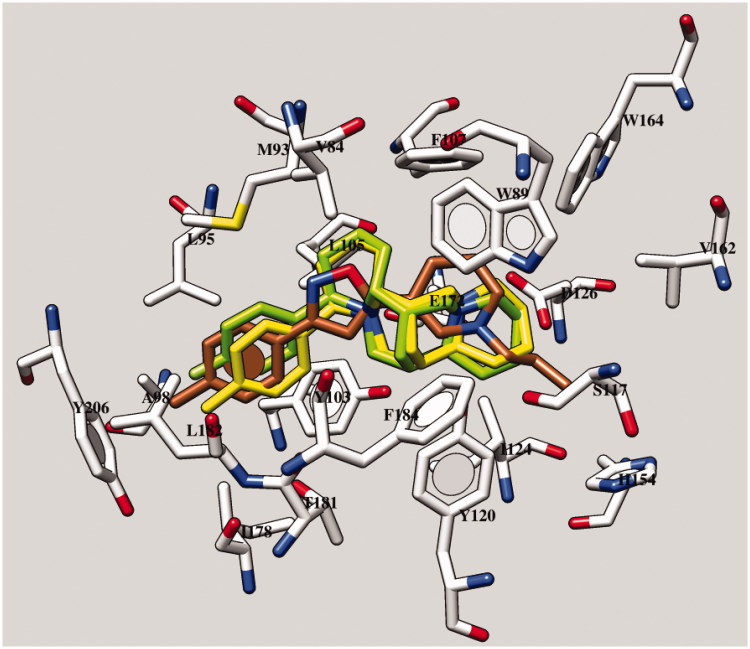
Docking mode of **7b** and of **7e** within the X-ray crystallographic data of the human sigma-1 receptor and of the ligand PD144418 (*C* atom; brown). The most important residues are labeled.

According to our calculations, the most promising compounds **7b** and **7e** (the *R* enantiomers proved to be preferred) overlapped the sparteine nucleus onto the tetrahydropyridine and oxazole moieties of the reference compound (Figure 7). As a consequence, the required contacts with D126 and E172 occurred. In addition, the overall sparteine architecture displayed hydrophobic contacts with V84, W89, F107, Y120, and F184, as exhibited by the reference compound oxazole ring and tetrahydropyridine moiety.

Moreover, the phenyl ring of **7b** and **7e** proved to highly mimic the role of the one of PD144418, establishing Van der Waals contacts with L95, A98, L182, and π − π stacking with Y103 and Y206. Interestingly, this kind of positioning proved to be allowed only when small lipophilic substituents decorate the phenyl ring *para* position, being projected toward a small cavity delimited by the aforementioned residues L95, A98, and L182. Indeed, the analogs **7e** and **7f** were endowed with a better inotropism profile than compound **7d**, bearing a less hydrophobic group.

On the contrary, derivatives characterized by a dehydrosparteine nucleus (**6b**, **6c**, **6e**, and **6f**) differently arranged within the sigma-1 receptor binding site, due to the more rigid and planar core. Consequently, they displayed a reversed docking mode with respect to PD144418, moving the phenyl ring and the dehydrosparteine cycle near the phenyl ring and the basic moiety of the reference compounds.

Concerning sparteine itself, it proved to partially overlap the related nucleus of the analog **7b**, exhibiting a quite comparable positioning. In particular, it moved much more in proximity of E172, displaying the required salt-bridge, while any contact with D126 is lacking. Conceivably, the absence of a phenyl substitution impairs the possibility to interact with the aforementioned residues A98, L182, and Y206.

On all these bases, the relevant sparteine derivatives may indeed act as efficient ligands for sigma-1 receptor.

## Conclusions

Ten sparteine derivatives (2-(4-substituted-phenyl)-2-dehydrosparteines and 2-(4-substituted-phenyl)sparteines) were tested for inotropic and chronotropic activity on reserpinized guinea pig atria. Four of them (**6b**, **6e**, **7b**, and **7f**) exhibited significant inotropic activity that, at the highest concentrations tested, was followed by negative inotropism or toxicity. Vice versa, compound **7e** (2-(4-tolyl)sparteine) exhibited a steep dose-depending positive inotropic activity up to the highest concentration (300 µM). At 10 µM, its activity was comparable with that of milrinone, but at 100 µM, the activity of **7e** was more than twice that of milrinone. Thus, in comparison with digoxin and milrinone, **7e** resulted less potent, but the most active.

The inotropic activity of compound **7b** was confirmed on electrically driven left atria from untreated guinea pig and was shown that its activity was not related to adrenergic stimulation or to inhibition of phosphodiesterase. A similar behavior was observed in the past for two, somewhat analogous, arylalkyl derivatives of cytisine^8^^,^^9^. Therefore, it is advanced the hypothesis that all the relevant compounds **6–9**, characterized by the combination of aromatic moieties with polycyclic alkaloidal frameworks, may share the same mechanism of action. On the base of some structural similarities between compounds **6–9** and sigma-1 receptor ligands, endowed with activity on cardiac muscle, it is supposed that the presently observed inotropic activity might be related to interaction with this receptor. Docking studies have, indeed, shown an excellent fitting of compounds **7b** and **7e** on the sigma-1 receptorial binding site.

Moreover, compound **7b** was shown to possess potent diuretic and saluretic activities, which might result very valuable in association to the positive inotropism to relieve symptoms of CHF.

Concluding, the 2-arylsparteine pharmacophore appears worthy of further investigation in order to develop novel agents for the treatment of CHF.
